# Characterization of the Composition and Immunoregulatory Activity of Wheat Cell Culture-Derived Polysaccharides

**DOI:** 10.3390/molecules31091540

**Published:** 2026-05-06

**Authors:** Alima Murtazina, Pol Rodríguez-Martínez, Dylan J. Crawshaw, Carme Caelles, Anel Tarabayeva, Elmira Bitanova, Nadezhda Ibragimova, Polina Mikshina, Tatyana Gorshkova, Gordon J. McDougall, Houria Boulaiz, Nazira Bishimbayeva, Annabel F. Valledor

**Affiliations:** 1 Department of General Immunology, Faculty of Medicine, Asfendyarov Kazakh National Medical University, Almaty 050012, Kazakhstan; murtazina.a@kaznmu.kz (A.M.); tarabaeva.a@kaznmu.kz (A.T.); bitanova.e@kaznmu.kz (E.B.); 2 Department of Anatomy and Human Embryology, Faculty of Medicine, University of Granada, 18016 Granada, Spain; hboulaiz@ugr.es; 3 Research Center “Bioscience Technologies”, Almaty 050057, Kazakhstan; 4 Institute of Biomedicine of the University of Barcelona (IBUB), 08028 Barcelona, Spain; prodriguezmartinez@ub.edu (P.R.-M.); crawshaw@recerca.clinic.cat (D.J.C.); ccaelles@ub.edu (C.C.); 5 Department of Cell Biology, Physiology and Immunology, School of Biology, University of Barcelona, 08028 Barcelona, Spain; 6 Department of Biochemistry and Physiology, School of Pharmacy and Food Sciences, University of Barcelona, 08028 Barcelona, Spain; 7 FRC Kazan Scientific Center, Kazan Institute of Biochemistry and Biophysics (KIBB), Russian Academy of Sciences, 420111 Kazan, Russia; nibra@yandex.ru (N.I.); mikshina@kibb.knc.ru (P.M.); gorshkova@kibb.knc.ru (T.G.); 8 Plant Biochemistry and Food Quality Group, Environmental and Biochemical Sciences Department, The James Hutton Institute, Invergowrie, Dundee DD2 5DA, UK; gordon.mcdougall@hutton.ac.uk; 9 Biosanitary Research Institute of Granada (ibs.GRANADA), 18012 Granada, Spain; 10 Biopathology and Regenerative Medicine Institute (IBIMER), 18016 Granada, Spain; 11 Research Institute for Problems of Biology and Biotechnology, Al-Farabi Kazakh National University, Almaty 050040, Kazakhstan

**Keywords:** polysaccharide, wheat cell culture, macrophage, cytokine, LPS

## Abstract

Plant polysaccharides can exert immunomodulatory activities. In this study we provided chemical characterization of wheat cell culture-derived polysaccharides (WCCPS) and assessed their capacity to modulate inflammatory responses in mouse macrophages. The total sample (T-010) contained arabinogalactans, arabinans, glucans and xyloglucans. Fractionation by anion-exchange chromatography rendered a bound acidic fraction (B-010) and an unbound neutral fraction (UB-010). The B-010 fraction was enriched in arabinogalactans and arabinans, with some galactans, homogalacturonans, and arabinoxylans. The neutral UB-010 fraction was composed of glucans and xyloglucans. None of the WCCPS preparations triggered cytokine production on their own, but each potentiated different macrophage responses to bacterial lipopolysaccharide (LPS). The total WCCPS in T-010 increased LPS-induced tumor necrosis factor-alpha (TNF-α) and interleukin (IL)-6 secretion, whereas the acidic arabinogalactan-rich fraction B-010 boosted IL-6 release and selectively upregulated nitric oxide synthase 2 (*Nos2*) and cholesterol 25-hydroxylase (*Ch25h*) expression in response to LPS. In contrast, the neutral UB-010 fraction enhanced IL-6 levels and induced *Nos2* expression without altering *Ch25h* expression. These results suggest that WCCPS can modulate distinct aspects of the inflammatory response, with their effects shaped by their composition and structural features. Future research will focus on elucidating the molecular mechanisms underlying the immunomodulatory activity of WCCPS.

## 1. Introduction

Polysaccharides are widely present in plants, algae, fungi and microorganisms [1]. In recent years, plant polysaccharides have attracted considerable attention as a source of natural biomacromolecules with a wide range of biological activities, including antioxidant, antitumoral, antiviral and immunomodulatory properties [2,3]. Plant polysaccharides display structural diversity, ranging from homopolysaccharides to heteropolysaccharides with different molecular weight (M_w_), branching patterns and monosaccharide composition. This structural complexity directly impacts their biological functions [4]. Importantly, plant polysaccharides are often characterized by a favorable safety profile, being relatively non-toxic and without significant adverse effects [5]. This is a distinct advantage over many synthetic compounds or even microbial polysaccharides, which may cause adverse reactions. High biocompatibility, bioavailability, and bioreactivity of plant polysaccharides [1] make them very attractive candidates for therapeutic development.

Macrophages play a central role in innate immunity, acting as phagocytes, antigen-presenting cells and major producers of cytokines that regulate inflammation and immune responses [6]. In response to bacterial lipopolysaccharide (LPS), macrophages produce various pro-inflammatory mediators, including inflammatory cytokines, prostaglandin E2 and nitric oxide. However, macrophages also play a key role in the negative regulation of inflammation through various mechanisms, including the production of anti-inflammatory cytokines [7]. Macrophages have been recognized as a primary cellular target for the immunomodulatory effects of plant polysaccharides [8].

Several plant polysaccharides exert immune-stimulating effects on macrophages by promoting cytokine secretion and generating reactive oxygen species and nitric oxide [9]. Despite the recognized broad immunomodulatory effects of plant polysaccharides, a comprehensive understanding of their differential effects on macrophage activation, particularly in different inflammatory contexts, remains an active area of research [9]. The complex structural diversity of polysaccharides suggests that their biological activities are likely to be highly specific and context-dependent.

Plant cell culture techniques provide promising means to modify the structural traits and production of bioactive metabolites by adjusting nutrient media and phytohormone types and concentrations [10,11,12,13]. Our previous investigations revealed that several wheat cell culture-derived polysaccharides (WCCPS) samples possess biological activity, including anti-proliferative effects on both differentiated and stem colorectal cancer cells [14,15]. The present study was designed with two objectives: firstly, to characterize the composition and structural features of the total and fractionated WCCPS samples; and secondly, to further assess the biological activity of these WCCPS, focusing on inflammatory responses in macrophages. To this end, mouse bone marrow-derived macrophages (BMDMs) were exposed to WCCPS either alone or in combination with bacterial LPS, and both basal and LPS-induced gene expression and cytokine secretion were evaluated. Our results suggest that WCCPS selectively modulate distinct components of the inflammatory response, an effect that is influenced by their chemical composition and structural characteristics.

## 2. Results

### 2.1. Structural Characterization of WCCPS

#### 2.1.1. Monosaccharide Composition

**Figure 1:**
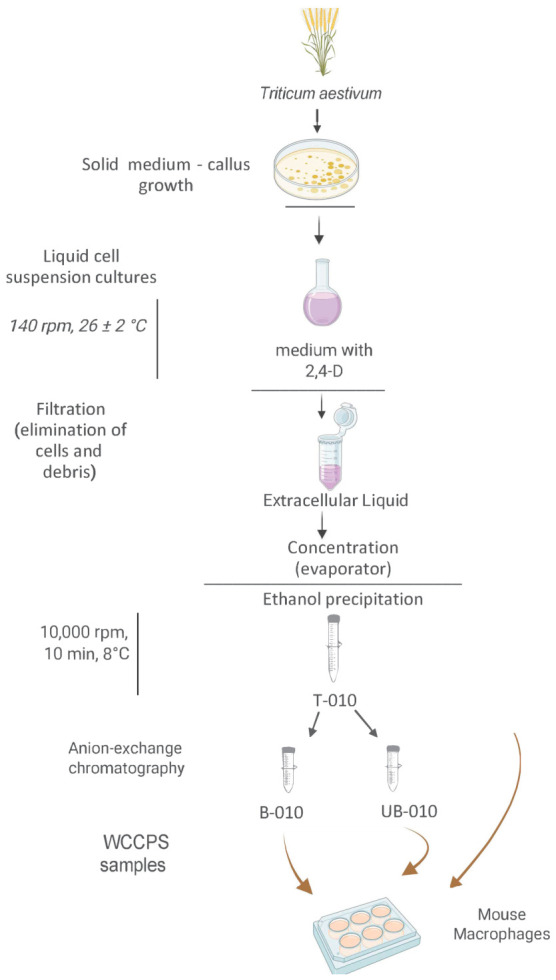
Preparation and application of wheat cell culture-derived polysaccharides (WCCPS). Callus derived from soft wheat (*Triticum aestivum*) were grown on solid agar nutrient medium. Subsequently, callus (200 mg) were used to obtain wheat cell suspensions in liquid nutrient medium supplemented with the phytohormone 2,4-dichlorophenoxyacetic acid (2,4-D) (5 mg/L). The cell suspensions were harvested, then filtered to remove cells and debris, and the liquid media was concentrated in a rotary evaporator. Extracellular polysaccharides were extracted with 70% ethanol, precipitated by centrifugation, and dissolved in ultrapure water. The T-010 sample consisted of the total extracellular polysaccharides. A portion of this preparation was subjected to further fractionation through anion-exchange chromatography, resulting in a bound fraction, B-010, and an unbound fraction, UB-010. The biological effects of WCCPS samples were evaluated in vitro using mouse macrophages. Image created in https://BioRender.com (accessed on 9 February 2026).

The WCCPS sample T-010 was obtained from the extracellular liquid of wheat (*Triticum aestivum*) cell suspension cultures grown in the presence of 2,4-dichlorophenoxyacetic acid (2,4-D) (Figure 1).

**Figure 2:**
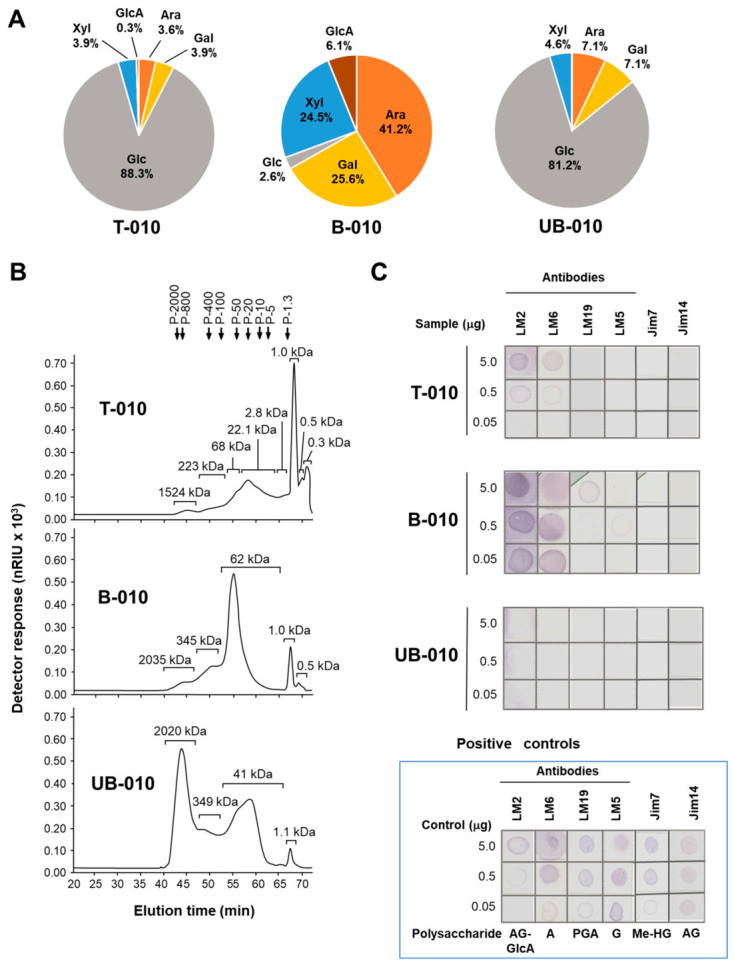
Monosaccharide composition, M_w_ distribution and structural elements of WCCPS in T-010, B-010 and B-010 samples. (**A**) Monosaccharide content in each sample was determined as indicated in Section 4. Relative monosaccharide contents are indicated as percentages. Ara, arabinose; Gal, galactose; Glc, glucose; GlcA, glucuronic acid; Xyl, xylose. (**B**) Elution profiles of the WCCPS samples after separation by size-exclusion chromatography. Pullulans of different molecular weight (M_w_) were used as standards, as indicated in Section 4. Estimated M_w_ of the elution peaks are shown in kDa. nRIU, nano-refractive index units; P, pullulans. (**C**) Immunodot binding assays of extracellular polysaccharides. Immunoblotting was performed as described in Section 4 using the following antibodies: LM2 specific to a carbohydrate epitope in arabinogalactan-protein containing β-linked GlcA [16,17], LM6 specific to α-(1,5)-L-Ara*f*_2–5_ [18,19], LM19 specific to de-esterified homogalacturonan [20], LM5 specific to β-D-(1,4)-Gal_3–5_ [21], Jim7 specific for methyl-esterified homogalacturonans [22], and Jim14 specific for differently branched β-1,6-linked galactan [23]. Standard polysaccharides with known structural elements were used as positive controls for each monoclonal antibody: A, arabinan; AG-GlcA, arabinogalactan with glucuronic acid residues; G, galactan; PGA, polygalacturonic acid; AG, arabinogalactan with branched galactan residues; me-HG, methyl-esterified homogalacturonan.

Analysis of the monosaccharide composition revealed that T-010 was composed predominantly of glucose (Figure 2A). Specifically, T-010 contained 88.3% glucose, along with arabinose (3.6%), galactose (3.9%), xylose (3.9%) and trace amounts of glucuronic acid. Two samples, B-010 and UB-010, were generated via the fractionation of the T-010 extract using anion-exchange chromatography [14]. The bound B-010 fraction contained only 2.6% glucose and was particularly rich in arabinose (41.2%), xylose (24.5%), galactose (25.6%), and notably acidic monosaccharides, glucuronic acid (6.1%) and galacturonic acid (trace). The unbound UB-010 fraction was glucose-rich (81.2%), with lower amounts of arabinose (7.1%), galactose (7.1%), and xylose (4.6%) but no uronic acids in contrast to B-010 and T-010 (Figure 2A).

#### 2.1.2. M_w_ Distribution of WCCPS

T-010 had eight peaks on separation by size-exclusion chromatography, four of which were pronounced, with retention times corresponding to M_w_ estimated at 1524 kDa, 68 kDa, 22.1 kDa, and 1 kDa against pullulan standards (Figure 2B).

The elution profile of fraction B-010 contained five peaks, with the major two having retention times corresponding to M_w_ estimated at 62 kDa and 1 kDa. Fraction UB-010 contained four peaks, the dominant ones having retention times corresponding to M_w_ estimated at 2000 kDa and 40 kDa (Figure 2B).

#### 2.1.3. Characterization of Polysaccharide Composition Using Immunological Assays

Dot blot analysis using antibodies specific against certain polysaccharide motifs indicated that the total sample T-010 did not react with Jim7 or Jim14, suggesting that it does not contain methyl-esterified homogalacturonans (Jim7-) [22] nor differently branched β-1,6-linked galactan (Jim 14-) [23]. Reaction with LM2 and LM6 (Figure 2C) suggested the presence of arabinogalactan associated with β-linked glucuronic acid residues (LM2+) [16,17] and oligomers of arabinose (LM6+) [18,19]. A positive reaction with LM2+ suggested the presence of arabinogalactan similar to Gum Arabic from acacia, a type II arabinogalactan (AG II), characterized by a higher uronic acid content compared to other arabinogalactans [24].

Dot blot analysis of the B-010 sample showed strong reactivity with LM2 (+++) (Figure 2C), which suggests that it contains high levels of AG II [16,17], which also contains glucuronic acid. Strong cross-reactivity with LM6 (+++) suggested the presence of linear oligomers of α-(1,5)-L-arabinan (minimum degree of polymerization of 5) [18,19]. Weak reactivity of the B-010 fraction with LM5 (+) [21,25] and LM19 (+) [20] suggested the presence of at least β-D-galactose 1,4-tetramers and non-esterified homogalacturonans.

No reactivity was observed for the UB-010 sample with any of the antibodies tested (Figure 2C), probably because acidic polysaccharides are retained in the B-010 fraction rather than in the UB-010 fraction. Indeed, the neutral UB-010 fraction did not contain uronic acids and was mainly composed of glucose with smaller amounts of arabinose, galactose and xylose, which suggests it may be predominantly composed of neutral glucans [26].

Taken together, the immunodot results and the monosaccharide composition analysis suggest that T-010 contains arabinogalactans, arabinans, glucans and xyloglucans. The bound B-010 acidic fraction was enriched in arabinogalactans, arabinans, with smaller contributions from galactans, homogalacturonans, and arabinoxylans. The unbound UB-010 fraction was composed presumably of neutral glucans and, possibly, xyloglucans. These differences in composition and structural features could lead to potentially different bioactivities.

### 2.2. WCCPS and Primary Macrophage Viability

**Figure 3:**
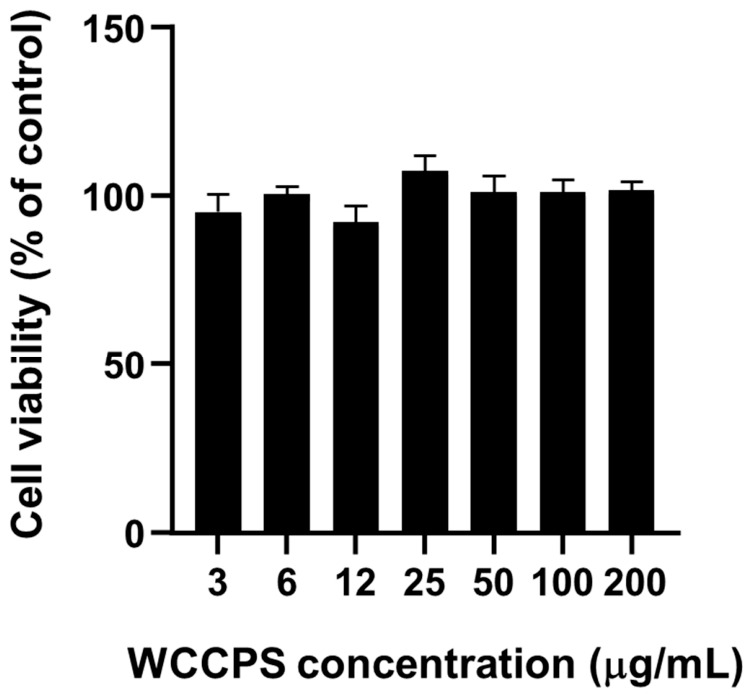
T-010 does not alter the viability of mouse macrophages. Bone marrow-derived macrophages (BMDMs) were differentiated for 7 days as described in Section 4. BMDMs (3 × 10^3^ cells/assay) were then treated during 24 h with the indicated concentrations of WCCPS (3–200 μg/mL) in DMEM-10% fetal bovine serum (FBS). Cell viability was evaluated using a 3-[4,5-dimethylthiazol-2-yl]-2,5 diphenyl tetrazolium bromide (MTT) assay as described in Section 4. Control cells were left untreated. The data are represented as relative cell viability compared to untreated control cells. Mean + SD. *n* = 3 biological replicates (from cells obtained from one male mouse). One-way ANOVA revealed no significant differences.

We evaluated whether WCCPS affected macrophage viability in vitro using a metabolic activity assay. BMDMs were treated for 24 h with the parental T-010 sample at different concentrations. T-010 had no significant effect on macrophage viability at the doses used in this study (Figure 3). As fractions B-010 and UB-010 were derived from the total T-010 preparation, we assumed that they would have no greater cytotoxicity than their parental sample.

### 2.3. Effects of WCCPS on Macrophage Pro-Inflammatory Responses

**Figure 4:**
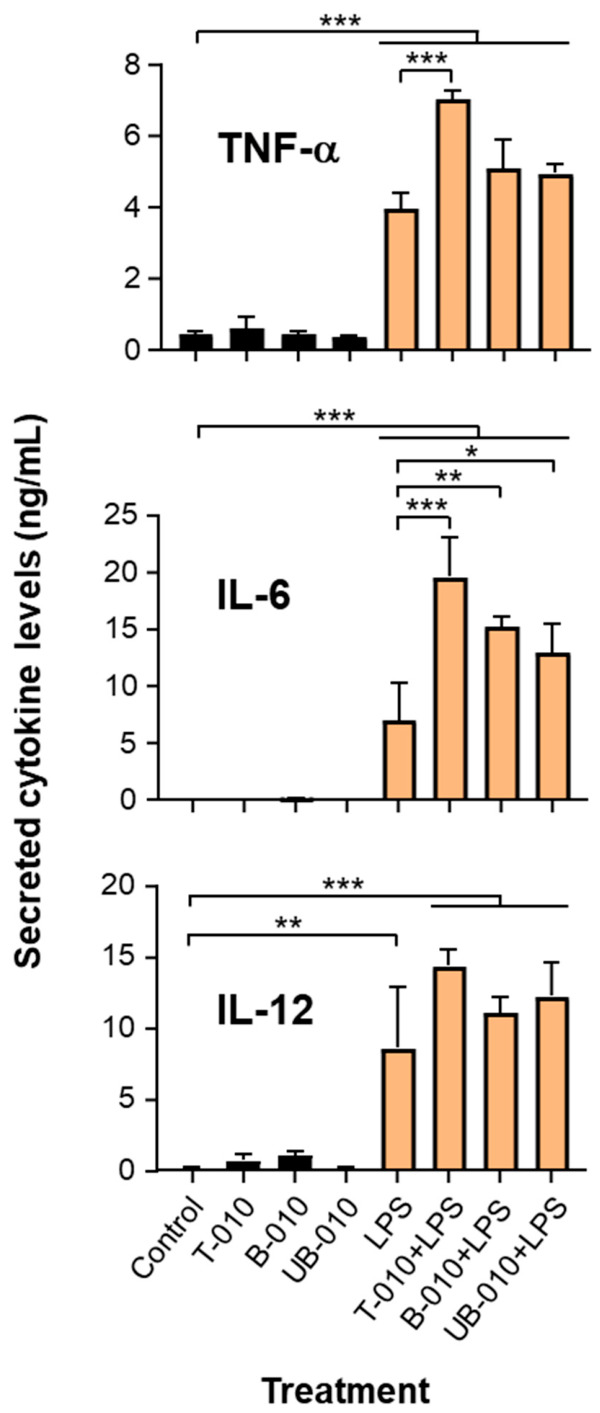
Effects of WCCPS on pro-inflammatory cytokine secretion. BMDMs (2 × 10^6^/well) were incubated with or without WCCPS (100 μg/mL in DMEM-10% FBS) for 18 h, followed by treatment with vehicle (phosphate buffer saline, PBS) or LPS (10 ng/mL) for 6 h. Control cells remained untreated. The supernatants were recovered and cytokine secretion was determined by ELISA as described in Section 4. Mean + SD. *n* = 2–3 biological replicates (using cells from two mice, one male and one female, in two separate experiments). One-way ANOVA-Tukey post hoc, * *p* < 0.05, ** *p* < 0.01, *** *p* < 0.001. IL, interleukin; TNF-α, tumor necrosis factor alpha.

BMDMs were treated for 18 h with WCCPS samples and then stimulated, or not, with bacterial LPS for 6 h. The secretion of the cytokines tumor necrosis factor alpha (TNF-α) and interleukins (IL)-6 and -12 was evaluated by ELISA. These cytokines represent key hallmarks of macrophage activation in response to LPS [27]. Notably, T-010 caused no significant increase in the secretion of TNF-α, IL-6 and IL-12 in the absence of LPS (Figure 4). The two T-010-derived fractions, B-010 and UB-010, showed a similar behavior to T-010, with no increase in inflammatory cytokine secretion (Figure 4). Interestingly, in LPS-stimulated cells, all WCCPS preparations significantly enhanced the secretion of IL-6, and sample T-010 was also effective in increasing the secretion of TNF-α (Figure 4). This indicates that different WCCPS preparations promoted selective cytokine secretion in the presence of additional inflammatory stimulators.

**Figure 5:**
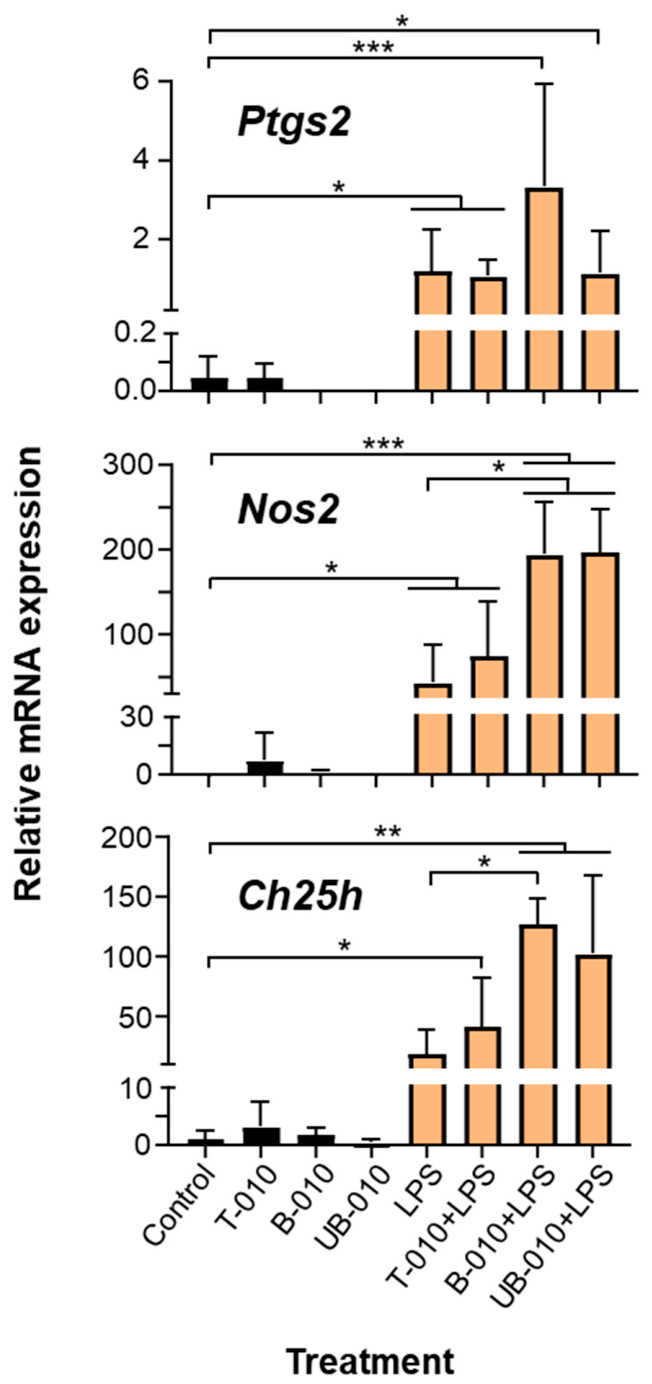
Effects of WCCPS on LPS-induced gene expression. BMDMs (2 × 10^6^/well) were incubated with or without WCCPS (100 μg/mL in DMEM-10% FBS) for 18 h, followed by treatment with vehicle (PBS) or LPS (10 ng/mL) for 6 h. Control cells remained untreated. The mRNA expression levels of several genes were determined via qRT-PCR and normalized by the expression levels of the ribosomal L14 protein. Mean + SD. *n* = 3–4 biological replicates (using cells from three mice, one male and two females, in two separate experiments). One-way ANOVA with Dunnet’s test for multiple comparisons, * *p* < 0.05, ** *p* < 0.01, *** *p* < 0.001. *Ch25h*, cholesterol 25-hydroxylase; *Nos2*, nitric oxide synthase 2; *Ptgs2*, prostaglandin-endoperoxide synthase 2.

Quantitative real-time PCR (qRT-PCR) was used to determine the effects of WCCPS on the expression of specific genes typically upregulated by LPS in macrophages, including the key pro-inflammatory enzymes prostaglandin-endoperoxide synthase 2 (PTGS2) and nitric oxide synthase 2 (NOS2), as well as cholesterol 25-hydroxylase (CH25H), which plays an important role in the antiviral response [28]. T-010 did not significantly promote the expression of these genes by itself or in combination with LPS (Figure 5). In contrast, fractions B-010 and UB-010 enhanced the LPS-induced expression of *Nos2* and, in the case of B-010, of *Ch25h* (Figure 5). Overall, this suggests that specific components in WCCPS may promote different aspects of the macrophage inflammatory response.

### 2.4. WCCPS Do Not Affect Tgfb Expression in Macrophages

**Figure 6:**
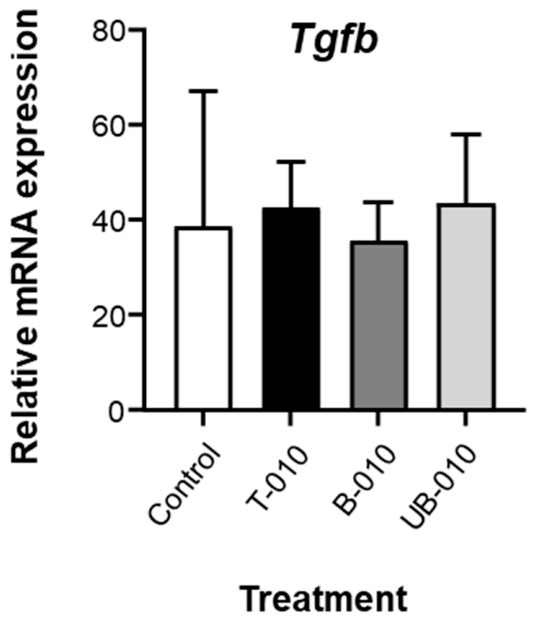
WCCPS do not downregulate *Tgfb* expression. BMDMs (2 × 10^6^/well) were incubated with WCCPS samples (100 μg/mL in DMEM-10% FBS) for 24 h. Control cells were left untreated. The mRNA expression levels of *Tgfb* were determined by qRT-PCR and normalized by the expression levels of the ribosomal L14 protein. Mean + SD. *n* = 3 biological replicates (using cells from three mice, one male and two females, in two separate experiments). One-way ANOVA revealed no significant differences.

Based on these pro-inflammatory activities of WCCPS, we assessed whether they could influence the basal expression of the anti-inflammatory cytokine transforming growth factor beta (TGFβ) in parallel. However, none of the WCCPS significantly affected *Tgfb* expression (Figure 6).

## 3. Discussion

Numerous studies have shown that plant polysaccharides can modulate innate and adaptive immune responses in multiple ways [9,29,30]. Several plant polysaccharides can activate macrophages through specific receptors and signaling cascades that promote inflammatory gene expression, whereas other plant polysaccharides exhibit anti-inflammatory properties [9,29]. Indeed, the immunoregulatory potential of polysaccharides has been closely linked to structural attributes such as their branching architecture, the presence of functional groups, the overall conformation, the specific types and ratios of monosaccharides, and the types of glycosidic bonds [30].

In this study, we assessed for the first time the structural characteristics of WCCPS and their potential to influence macrophage activities in basal conditions and during LPS-induced inflammation. The combined results of size-exclusion chromatography and dot blot analysis indicate different characteristics after fractionation. T-010 was a heterogenous mixture of arabinogalactans, arabinans, glucans and xyloglucans spanning a broad M_w_ range; B-010 contained medium- and low-M_w_ polysaccharides enriched in acidic pectic/AG II components; and UB-010 was predominantly composed of high-M_w_ polysaccharides rich in neutral glucan/xyloglucan components. The effects on macrophage functional responses suggest that these variations may support diverse bioactivities. An enhancement in LPS-induced IL-6 and TNF-α production was observed in macrophages treated with T-010. While IL-6 is widely known as a pro-inflammatory cytokine and a key mediator of the acute phase response, it can also have a fine-tunning role by suppressing the levels of other pro-inflammatory cytokines and chemokines during inflammation [31]. This cytokine can also promote the accumulation of myeloid-derived suppressor cells in the context of cancer and some types of infection [32].

Given the absence of an effect without LPS stimulation T-010 appears to function not as a primary pro-inflammatory agonist, but rather as a selective modulator that enhances an existing inflammatory signal. A similar effect has been shown for soluble β-1,3-glucan, which induced minor amounts of TNF-α and IL-6 in whole blood samples, but strongly primed LPS stimulation of these cytokines [33]. Several pathogen recognition receptors have been reported to recognize β-glucans in macrophages, including Dectin-1 and complement receptor 3 [34]. Whether the polysaccharides present in the T-010 preparation engage these receptors to co-stimulate the LPS response remains to be determined. Future studies should also examine whether these polysaccharides amplify macrophage activation by increasing the expression or sensitivity of LPS-recognition components such as cluster of differentiation 14 (CD14), myeloid differentiation protein 2 (MD-2), and toll-like receptor 4 (TLR4), by modulating their intracellular signaling, or by enhancing LPS uptake. Notably, the fractions B-010 and UB-010 augmented LPS-induced production of IL-6, but not of TNF-α. This observation suggests that the combination of polysaccharides present in T-010 is necessary for the positive effect that leads to increased TNF-α release by LPS, and that their fractionation compromises this capacity. Similarly, T-010 demonstrated greater efficacy in suppressing the proliferation of a colon cancer cell line compared to its individual fractions [14]. In contrast, both B-010 and UB-010 fractions promoted the expression of *Nos2* in response to LPS stimulation, an effect not observed with the parental T-010 preparation. B-010 also significantly enhanced *Ch25h* expression following LPS exposure. NOS2 is responsible for the production of nitric oxide, which plays a crucial role in the antimicrobial defense [35], whereas CH25H, via synthesis of 25-hydroxycholesterol, serves as a host factor with antiviral potential implicated in the regulation of virus entry [28]. The fact that B-010 significantly upregulated *Nos2* and *Ch25h* expression alongside IL-6 secretion, but not TNF-α, during the response to LPS suggests that this fraction may selectively activate host defense mechanisms without triggering the full inflammatory response. UB-010 partially mirrored the effects of B-010 by enhancing IL-6 secretion and inducing *Nos2* expression upon LPS stimulation, but it did not significantly alter *Ch25h* expression, indicating a more limited modulatory capacity.

These findings suggest that specific polysaccharides enriched in these fractions may contribute to the selective enhancement of certain responses triggered by LPS. The shared effects of both B-010 and UB-010 likely reflect common structural motifs capable of engaging similar macrophage receptors. In contrast, the selective induction of *Ch25h* by B-010 may stem from differences in monosaccharide composition and structural features, such as the higher abundance of AG II and uronic acids in B-010. Arabinogalactans have been shown to promote pro-inflammatory macrophage polarization, enhancing nitric oxide and cytokine production and accelerating antimicrobial responses. They can also activate the complement cascade [36,37,38]. Dietary supplementation with Gum Arabic arabinogalactan upregulated the expression of the antimicrobial peptide cathelicidin in macrophages [39], although its in vivo effects may be mediated by its fermentation into short chain fatty acids by the gut microbiota [40]. Whether the high arabinogalactan content of B-010 contributes to enhancing selective aspects of the macrophage response to LPS remains to be elucidated. Clarifying these mechanisms will require dedicated receptor-ligand interaction studies and more detailed structural analyses. It is also important to note that while LPS strongly induces NOS2 expression in murine models, human macrophages exhibit limited NOS2 induction in response to LPS [34]. The ability of B-010 and UB-010 to promote NOS2 expression in an LPS-co-stimulated context warrants further investigation in human macrophages, as they may offer targeted strategies to activate this key antimicrobial pathway in a typically hyporesponsive system.

None of the fractions altered *Tgfb* expression, consistent with many plant polysaccharides that selectively engage pro-inflammatory pathways without modulating anti-inflammatory signals in short-term assays [9,41]. These patterns mirror those in dietary polysaccharides, where uronic acid content and M_w_ positively correlate with macrophage immunostimulatory potency [42,43]. However, they differ from the behavior of certain suppressive acidic pectins whose unbranched structures can inhibit LPS-driven activation [42]. Such insights underscore the potential of WCCPS as targeted immunomodulators, warranting further studies on receptor interactions and in vivo efficacy.

In summary, our study suggests that a total WCCPS preparation and its fractions derived by ion-exchange chromatography differ markedly in chemical composition, structural features, and M_w_, resulting in distinct modulatory effects on macrophages during inflammation. These observations align with recent evidence showing that processes such as fermentation, enzyme-assisted extraction, or fractionation can alter the composition and M_w_ of polysaccharides, thereby reshaping their immunomodulatory properties [44,45,46,47]. Future studies should aim to identify the pattern-recognition receptors involved in WCCPS sensing by macrophages and to elucidate the mechanisms that drive the selective amplification of specific inflammatory responses. In parallel, further work is necessary to more precisely define the chemical structures and glycosidic linkages of WCCPS through advanced technologies, including nuclear magnetic resonance and infrared spectroscopies. This study was conducted using mouse BMDMs, a highly reproducible model that enables the generation of large numbers of macrophages. However, future experiments evaluating the relevance of these findings in in vivo models of inflammation and in human macrophages will be essential to establish their physiological and translational significance.

## 4. Materials and Methods

### 4.1. Extraction and Fractionation of WCCPS

Wheat cell suspension cultures were cultivated using callus tissues derived from soft wheat *Triticum aestivum* Kazakhstanskaya 10 variety (kindly provided by Pr. R.A. Urazaliyev and Pr. I.A. Nurpeisov, Kazakh Research Institute of Agriculture and Plant Growing, Almaty, Kazakhstan, https://adilet.zan.kz/rus/docs/V090005759_, KZ9210, accessed on 1 April 2026), as described in previous studies [14,48]. Briefly, 200 mg of callus tissue were cultured in 30 mL of liquid Murashige and Skoog medium (Sigma–Aldrich, St. Louis, MO, USA) [49], supplemented with the phytohormone 2,4-D (5 mg/L) (Sigma–Aldrich) at 26 ± 2 °C for a 16 h light/8 h dark photoperiod with continuous agitation (140 rpm) for one week. The cell suspensions were harvested and filtered to remove cells and debris. The liquid media were recovered and then concentrated using a rotary evaporator (RV3V; IKA, Staufen, Germany). Extracellular polysaccharides were precipitated using 70% ethanol and collected via centrifugation at 10,000 rpm for 10 min at 8 °C. The total amount of carbohydrate content was determined using the Dubois method [50] in a SmartSpec spectro-photometer (Bio-Rad, Hercules, CA, USA). The T-010 sample was the total extracellular polysaccharides obtained from a wheat cell suspension culture grown in the presence of 2,4-D. A portion of this preparation was subjected to further purification through anion-exchange chromatography using a DEAE-Sepharose column (Sigma–Aldrich) as previously reported [14,51], resulting in two separate fractions—an acidic (bound, B-010) and a basic (unbound, UB-010). WCCPS were dissolved in milliQ water for stock solutions. In each experiment, the stock solutions were further diluted in DMEM (Biowest, Nuaillé, France) to obtain the desired concentrations. These stock solutions were tested for the presence of endotoxin using a Pierce™ Chromogenic Endotoxin Quant Kit (Thermo Fisher Scientific, Waltham, MA, USA) with a lower detection limit of 0.01 EU/mL. No detectable levels of endotoxins were found in any of the WCCPS samples used in this study.

### 4.2. Determination of Monosaccharide Composition

The monosaccharide profile of the polysaccharides in each sample was determined by acid hydrolysis followed by analysis using high-performance anion exchange chromatography [52]. For this purpose, the samples were hydrolyzed with 1 mL of 2 M trifluoroacetic acid (Sigma–Aldrich) at 120 °C for 2 h. After centrifugation at 10,500× *g* for 5 min, the hydrolysates were dried under vacuum using a Speed-Vac system (Genevac, miVac Duo, Ipswich, UK) and then dissolved in ultrapure water. The monosaccharide composition was determined based on calibration curves of standard compounds separated on a CarboPAC PA20 chromatography column (Thermo Fisher Scientific) and recorded by pulsed amperometric detection in a Dionex ICS-5000 system (Thermo Fisher Scientific).

### 4.3. Determination of M_w_ of WCCPS by Gel-Permeation Chromatography/Size-Exclusion Chromatography (GPC/SEC)

Separation was carried out on sequentially connected Shodex columns (Showa Denko, Tokyo, Japan): OHpak SB-G (6.0 × 50 mm, precolumn), OHpak SB-806M HQ (8.0 × 300 mm) and OHpak SB-804 HQ (8.0 × 300 mm) in 0.1 M NaOAc buffer with 0.02% NaN3, pH 5.5 (flow rate 0.5 mL/min, column temperature 40 °C, loop volume 50 μL). Detection was carried out using an Agilent 1260 Infinity refractometric detector (Agilent Technologies, Santa Clara, CA, USA) at 35 °C. Dextran 2000 kDa (Sigma–Aldrich), pullulans 380, 186, 100, 48, 23.7, 12.2, 5.8 kDa (Showa Denko) with a low polydispersity index (1.09–1.19) and D(+)-glucose (Merck, Darmstadt, Germany) were used as standards. Agilent GPC/SEC software version 1.2. (Agilent Technologies) was used for data processing.

### 4.4. Determination of Structural Elements Included in the Composition of Extracellular Polysaccharides Using Immunodot Analysis

For each determination, an aliquot of each sample (0.02–5 μg in 1.5–2 μL), was applied to a nitrocellulose membrane (0.2 μm, Sigma–Aldrich) in carbonate-bicarbonate buffer (0.05 M, pH 9.6). The membranes with the applied samples were air-dried for 30 min and washed for 5 min in phosphate buffer saline (PBS) with 0.05% Triton (PBST), then blocked for 40 min in 3% bovine serum albumin (BSA) in PBS and washed with PBST for 5 min. Then, the membranes were incubated with primary monoclonal antibodies (diluted 1:30) in PBST for 50 min. The monoclonal antibodies used in this study have been previously described [25]: LM2 is specific for a carbohydrate epitope in arabinogalactan-protein containing β-linked GlcA [16,17], LM6 is specific for α-(1,5)-L-Ara*f*_2–5_ [18,19], LM19 is specific for de-esterified homogalacturonan [20], Jim7 is specific for methyl-esterified homogalacturonans [22], Jim14 is specific for differently branched β-1,6-linked galactan [23], LM5 is specific for β-D-(1,4)-Gal_3–5_ [21]. The membranes were washed three times for 10 min with 0.1% BSA in PBS, and then incubated with the conjugate anti-rat IgG-Biotin (1:9000) in PBST for 50 min. The membranes were then incubated with conjugate 2 (streptavidin-alkaline phosphatase (Silex, Asbest, Russia) and developed using a nitroblue tetrazolium/5-bromo-4-chloro-3-indolyl phosphate kit (Silex) according to the manufacturer’s recommendations. The membranes were then washed in distilled water for 5 min, dried, and scanned. The reactivity in the absence of primary antibodies was negative.

The control for each monoclonal antibody was a standard polysaccharide with a known composition and reactivity to rat antibodies [23]: LM2—Gum Arabic from acacia (Sigma–Aldrich); LM5—Potato galactan (Megazyme, Bray, Co. Wicklow, Ireland); LM6—Linear arabinan from sugar beet (Megazyme); LM19—Polygalacturonic acid (Megazyme); Jim7—Homogalacturonan methyl-esterified, fraction of KIBB (Kazan, Russia); Jim 14—arabinogalactan (Megazyme). For each individual membrane, a control was applied at a concentration similar to the corresponding sample.

### 4.5. Animals

C57BL/6 mice were purchased from Envigo (Indianapolis, IN, USA) and raised as a colony in our animal facility. The mice were provided with water and chow diet ad libitum. Both biological sexes (equal ratio) were used in this study. All of the protocols requiring animal manipulation have been approved by the ethical committee for experimentation with animals at Universitat de Barcelona (number 222/19, approved on 1 December 2021) following the standard ethical regulations and meeting quality and experimental requirements of current applicable National (RD 53/2013, article 38) and European legislation (RD 53/2013 Council Directive; 2010/63/UE; Order 214/1997/GC).

### 4.6. Obtention of Primary Murine Macrophages

BMDMs were obtained from 6- to 8-week-old C57BL/6 mice as described [53]. Briefly, the femurs and tibias were dissected and placed in PBS. The bone marrows were flushed out with DMEM (4.5 g/L glucose, with L-glutamine, without sodium pyruvate) (Biowest) using 25G needles (Terumo, Tokyo, Japan). Clumps were disaggregated through a 25G needle and the cells were plated in 150 mm Petri dishes (Deltalab, Rubí, Spain). Bone marrow precursors were differentiated into macrophages during 7 days in DMEM supplemented with 20% heat-inactivated fetal bovine serum (FBS) (Merck), 30% L-cell conditioned medium, and 1% penicillin/streptomycin (Capricorn Scientific, Ebsdorfergrund, Germany) at 37 °C.

### 4.7. Cell Viability Assay

Cell viability was tested using a 3-[4,5-dimethylthiazol-2-yl]-2,5 diphenyl tetrazolium bromide (MTT) colorimetric assay (Merck) [54]. Briefly, pooled BMDMs were plated in 96-well plates (3 × 10^3^ cells/well) and incubated with different concentrations of WCCPS in DMEM supplemented with 10% FBS and 1% penicillin/streptomycin at 37 °C. After 24 h, the medium was removed, and the cells were incubated with MTT reagent (50 μL) for 3 h at 37 °C. Next, 100 μL of dimethyl sulfoxide (DMSO) was added to each well to dissolve the formazan product. The absorbance was measured at 570 nm with an Infinite M200 luminometer (TECAN, Männedorf, Switzerland).

### 4.8. RNA Extraction, Reverse Transcription and Quantitative Real-Time PCR Analysis

BMDMs were plated in 6-well plates (2 × 10^6^ cells/plate) in DMEM-10% FBS. The cells were treated with WCCPS (100 μg/mL) for 18 h and then stimulated with LPS (10 ng/mL) or vehicle (PBS) for 6 h. Control cells were left untreated. Total RNA was extracted using QIAzol (Qiagen, Venlo, Netherlands) as recommended by the manufacturer. Briefly, the cells were lysed in 250 μL QIAzol lysis reagent by pipetting up and down. The samples were extracted with chloroform (60 μL/sample) and then centrifuged for 15 min. For each sample, the aqueous phase was transferred to a new tube. Total RNA precipitation was performed by adding 125 μL isopropanol and incubation at −80 °C overnight. The samples were centrifuged for 10 min and the RNA pellets were washed twice with 75% ethanol. The pellets were air-dried and resuspended in RNase-free water.

For cDNA synthesis, 1 μg total RNA was incubated with 0.5 μg oligo(dT)_15_ primers (Merck) in milliQ water at 70 °C for 5 min. After cooling the samples on ice, the samples were supplemented with M-MLV RT buffer (1X final concentration), 10 mM dNTPs, and 200 U M-MLV Reverse transcriptase RNase H Minus, Point Mutant (Promega, Madison, WI, USA). First-strand synthesis of cDNA was allowed to proceed at 40 °C for the initial 10 min and between 40 and 55 °C for the next 50 min. The reaction was inactivated at 70 °C for 15 min. The samples were diluted 1:4 in milliQ water and kept at −20 °C until subsequent gene expression analysis.

qRT-PCR was performed using NZYSupreme qPCR Green Master Mix (NZYTech, Lisbon, Portugal), 2 μL of each diluted cDNA sample and 250 nM of each primer. The primers used for qRT-PCR are displayed in Table 1. Real-time monitoring of PCR amplification was performed using the CFX384 Real-Time System (Bio-Rad). Following an initial denaturation at 95 °C for 10 min, the samples were subjected to 35 amplification cycles of 95 °C-30 s, 60 °C-30 s, and 72 °C-30 s, after which a melting curve analysis was performed from 65 °C to 95 °C. The data were expressed as relative mRNA levels normalized to the expression values of the gene coding for the ribosomal protein L14.

**Figure 7:**
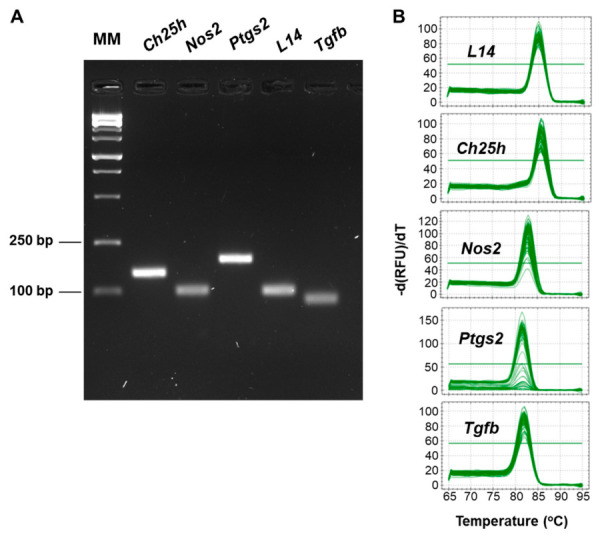
Primer pair specificity. (**A**) cDNA obtained from BMDMs was amplified by PCR using the pairs of primers listed in Table 1. cDNA from BMDMs stimulated with LPS (100 ng/mL, 6 h) was used to amplify *Ch25h*, *Nos2* and *Ptgs2*, whereas cDNA from untreated BMDMs was used to amplify *L14* and *Tgfb*. The resulting amplicons were separated by electrophoresis on a 2% agarose gel containing Sybr Safe. (**B**) Melting peaks obtained for each primer pair after completion of the qRT-PCR assays. bp, base pairs; MM, molecular marker; RFU, relative fluorescence units; T, temperature.

The specificity of each primer pair (each at 250 nM) was evaluated by standard PCR using cDNA (0.1 μg) from BMDMs as template and Supreme NZYTaq II Green Master Mix (NZYTech). For this assay, cDNA samples were denatured at 95 °C for 4 min, followed by 35 amplification cycles consisting of 94 °C for 30 s, 60 °C for 30 s, and 72 °C for 1 min. After cycling, the reaction proceeded to a final extension at 72 °C for 10 min. The samples were then separated by electrophoresis and stained with SYBR Safe (Invitrogen, Carlsbad, CA, USA). Each primer set yielded a distinct amplification product (Figure 7A). In addition, melting curve analysis was performed after qRT-PCR, confirming that each primer pair generated a single amplification product (Figure 7B).

### 4.9. Enzyme-Linked Immunosorbent Assay (ELISA)

Supernatants were collected after treating macrophages with various stimuli and diluted 1:5 in PBS. The levels of secreted TNF-α, IL-6 and IL-12 were measured using specific sandwich ELISA kits (PeproTech, #900-K54, #900-K50 and #900-K97, respectively). Briefly, 96-well plates (Maxisorp NUNC^TM^, Thermo Fisher Scientific) were coated with capture antibody (1 μg/mL in PBS) for 15 h, blocked with 1% BSA in PBS for 1 h, incubated sequentially with the samples of interest or a standard curve for 2 h, with the detection antibody (1 μg/mL in 0.1% BSA-PBST) for 2 h and with an avidin-HRP conjugate (1:2000) for 30 min. All incubations were performed at room temperature and the wells were washed 3 times in PBST after every step. The wells were then incubated with o-phenylenediamine dihydrochloride in citrate-phosphate buffer (pH = 4.2, 70 mM) supplemented with H_2_O_2_. The absorbance was monitored at room temperature every 5 min at 450 nm with a TECAN Infinite 200 Plate Reader (Tecan).

### 4.10. Statistical Analysis

GraphPad Prism 8.0 software was used to perform all statistical analyses. All datasets had normal distribution and were analyzed using one-way ANOVA. To make different experiments comparable, the data were normalized using the following procedure. The intensity of each experiment (ie) was calculated by determining the mean value of gene expression between the negative and positive controls. The intensities of separate experiments were normalized by the mean intensity value of all the experiments (im) and, for each experiment, the resulting normalization factor (im/ie) was multiplied by the expression levels of all the samples in that experiment. There were no data excluded from the analysis.

### 4.11. Randomization and Blinding

The assignment of treatments to wells were not subjected to randomization or to strategies trying to minimize potential confounders. The study was not conducted in a blind manner.

## 5. Conclusions

This study shows for the first time that extracellular polysaccharides from wheat cell cultures are a promising source of immunomodulatory compounds. The WCCPS in this study did not activate macrophages directly but enhanced LPS-induced responses, indicating a modulatory rather than a primary pro-inflammatory effect. The total WCCPS sample (T-010) amplified LPS-induced cytokine production (IL-6 and TNF-α), whereas its fractions revealed more specialized activities. The acidic B-010 fraction selectively increased IL-6, *Nos2*, and *Ch25h*, while the neutral UB-010 fraction enhanced IL-6 and *Nos2* but not *Ch25h*, reflecting a narrower functional profile. These selective activities distinguish WCCPS from conventional broad-spectrum inflammatory agents and support their potential for therapeutic development.

Future work should identify the receptors and signaling pathways involved and further resolve the structural features responsible for these effects. Overall, plant cell cultures offer a controlled platform for generating bioactive polysaccharides with potential applications as vaccine adjuvants or selective immunomodulatory agents in infectious disease and cancer.

## Figures and Tables

**Table 1:** Primers used for qRT-PCR analysis. Primer pairs (forward and reverse) for each of the genes analyzed in this study. The expected amplicon size is indicated in base pairs (bp).

Gene	Forward Primer (5′→3′)	Reverse Primer (5′→3′)	Size (bp)
*Ch25h*	TGCTACAACGGTTCGGAGC	AGAAGCCCACGTAAGTGATGAT	148
*L14*	TCCCAGGCTGTTAACGCGGT	GCGCTGGCTGAATGCTCTG	101
*Nos2*	GCCACCAACAATGGCAACA	CGTACCGGATGAGCTGTGAATT	103
*Ptgs2*	ATTCTTTGCCCAGCACTTCA	GGGATACACCTCTCCACCAA	193
*Tgfb*	GAGCCCGAAGCGGACTACTA	TGGTTTTCTCATAGATGGCGTTG	82

## Data Availability

The original contributions presented in this study are included in the article. The raw data supporting the conclusions of this article will be made available by the authors on request. Inquiries can be directed to the corresponding authors.

## Abbreviations

The following abbreviations are used in this manuscript:

2,4-D	2,4-dichlorophenoxyacetic acid
AG II	type II arabinogalactan
BMDM BSA	bone marrow-derived macrophage bovine serum albumin
CH25H/*Ch25h*	cholesterol 25-hydroxylase
IL	interleukin
LPS	lipopolysaccharide
M_w_	molecular weight
NOS2/*Nos2* PBS PBST	nitric oxide synthase 2 phosphate buffer saline phosphate buffer saline with 0.05% Triton
PTGS2/*Ptgs2*	prostaglandin-endoperoxide synthase 2
TGFΒ/*Tgfb*	transforming growth factor-beta
TNF-α	tumor necrosis factor-alpha
WCCPS	wheat cell culture-derived polysaccharides
